# Associations of serum Hcy, SAP, NfL, and carotid plaque Crouse score with white-matter hyperintensities in older patients with cerebral small-vessel disease

**DOI:** 10.3389/fneur.2026.1977360

**Published:** 2026-10-21

**Authors:** Bo Ren, Yuanyuan Ma, Wenjing Mao, Xiaokun Wu

**Affiliations:** Fourth Department of Neurology, North China University of Science and Technology Affiliated Hospital, Tangshan, China

**Keywords:** carotid plaque Crouse score, cerebral small vessel disease, homocysteine, neurofilament light chain, serum amyloid P component, white matter hyperintensities

## Abstract

**Background and purpose:**

This study aimed to investigate the correlations of serum homocysteine (Hcy), serum amyloid P component (SAP), neurofilament light chain (NfL), and carotid plaque Crouse score with white matter hyperintensities (WMH) in elderly patients with cerebral small-vessel disease (CSVD).

**Methods:**

A total of 382 MRI-confirmed elderly CSVD patients hospitalized in the Department of Neurology, North China University of Science and Technology Affiliated Hospital from July 2022 to June 2024 were enrolled. Patients were divided into WMH-positive and WMH-negative groups, and WMH-positive cases were further stratified into mild, moderate, and severe subgroups via the Fazekas scale. We compared serum Hcy, SAP, NfL levels and carotid plaque Crouse score among groups. Spearman rank-order correlation was used to analyze the relationships between the above indicators and WMH severity. Binary logistic regression identified independent correlates for WMH occurrence, while ordinal logistic regression together with the proportional-odds (parallel-lines) test to explore independent correlates of WMH severity. ROC curve analysis evaluated the diagnostic performance of each indicator. A two-sided *p* < 0.05 was defined as statistically significant.

**Results:**

Age, serum Hcy, SAP, NfL levels and carotid plaque Crouse score were significantly higher in the WMH-positive group (all *p* < 0.05). All indicators increased stepwise with the aggravation of WMH lesions, and pairwise comparisons showed significant differences among mild, moderate and severe subgroups (all *p* < 0.05). Spearman analysis confirmed that all indicators were positively correlated with WMH severity (all *p* < 0.001). After adjusting for confounding factors, age, serum Hcy, SAP, NfL and carotid plaque Crouse score were independently associated with WMH occurrence and severity (all *p* < 0.05). All indicators showed certain diagnostic value for CSVD-related WMH; SAP had the optimal efficacy, Crouse score and Hcy presented high specificity, while NfL showed the weakest discriminatory ability.

**Conclusion:**

Serum Hcy, SAP, NfL and carotid plaque Crouse score were independently associated with WMH occurrence and severity in elderly CSVD patients. These indicators exhibit diagnostic performance for CSVD-related WMH, with SAP showing the best diagnostic value. They can provide reliable references for early clinical screening and severity evaluation of WMH. Further studies are required to clarify the underlying pathophysiological mechanisms of these associations.

## 1 Introduction

Cerebral small vessel disease (CSVD) is a clinical-imaging-pathological syndrome caused by pathological alterations of intracranial small arteries, arterioles, capillaries, and small veins. It frequently manifests as cognitive impairment, gait disturbance, urinary dysfunction, and other clinical features. Characterized by insidious onset and slow progression, CSVD has become a major neurological disorder threatening the health of middle-aged and older adults. The diagnosis of CSVD largely relies on neuroimaging examinations, among which white matter hyperintensities (WMH) represent one of its most representative imaging biomarkers. Cumulative evidence indicates that WMH burden is significantly associated with cognitive decline, motor dysfunction, and mood disturbances such as depression. WMH not only severely impairs patients’ quality of life but also imposes a substantial burden on families and society.

The pathogenesis of WMH is multifactorial, involving multiple pathophysiological processes including chronic cerebral ischemia, blood–brain barrier disruption, neuroinflammation, and axonal injury (1). Therefore, identifying potential biomarkers underlying these diverse mechanisms is critical for etiological stratification, risk prediction, and individualized intervention for WMH. Homocysteine (Hcy), a key intermediate in human methylation and energy metabolism, exerts neurotoxic effects when elevated and impairs vascular endothelial function, promoting the development of atherosclerosis and microvascular lesions (2). Serum amyloid P component (SAP), an evolutionarily highly conserved cyclic pentameric plasma glycoprotein belonging to the pentraxin family, modulates immune responses and inhibits elastase activity. Studies have demonstrated that SAP participates in and accelerates inflammatory cascades by upregulating pro-inflammatory cytokines and activating the complement system (3). Neurofilament light chain (NfL) is a core structural component of the neuronal axonal cytoskeleton. It is released into cerebrospinal fluid and peripheral blood upon axonal damage, serving as a valuable biomarker for neurodegeneration and white-matter microstructural injury. Although cerebrospinal fluid NfL exhibits high sensitivity and specificity, its invasive collection limits routine clinical application. By contrast, serum or plasma NfL measurement is more clinically feasible and has been adopted in numerous studies to evaluate CSVD-related neuronal injury (4). The carotid plaque Crouse score is a classic ultrasonographic metric for quantifying total carotid atherosclerotic plaque burden. It objectively evaluates overall atherosclerotic load by summing the maximum thickness of discrete plaques and is widely used to assess the severity of large-vessel disease. Progressive carotid atherosclerosis may trigger chronic ischemic injury in distal cerebral white-matter regions (5).

Existing studies have separately explored the associations of Hcy, NfL, and the carotid plaque Crouse score with CSVD (2–5), whereas data regarding SAP and WMH remain scarce. Comprehensive studies integrating these multi-dimensional indicators and performing stratified analyses according to WMH severity among older adults are currently lacking. Our research group previously explored relevant clinical characteristics of this disease based on different patient cohorts (6). The present study further supplements and validates the related findings using an independent dataset. In this study, 382 consecutively hospitalized older patients with CSVD who underwent brain magnetic resonance imaging (MRI) were enrolled. Complete clinical, imaging, and serological data were collected. This study aimed to analyze the correlations of serum Hcy, SAP, NfL levels, and carotid plaque Crouse score with WMH lesions. The findings may further elucidate the potential mechanisms underlying white-matter injury in CSVD and provide evidence-based references for early screening, clinical diagnosis, and individualized prevention and treatment of CSVD.

## 2 Materials and methods

### 2.1 Study population

This study was reviewed and approved by the Ethics Committee of North China University of Science and Technology Affiliated Hospital (approval No. 20220605009) and conducted in strict accordance with the principles of the Declaration of Helsinki.

A total of 382 older adult patients with CSVD were consecutively enrolled from inpatients admitted to the Department of Neurology, North China University of Science and Technology Affiliated Hospital between July 2022 and June 2024. The diagnosis of CSVD was established according to the 2020 Chinese Guidelines for the Diagnosis and Treatment of Cerebral Small-Vessel Disease (2), and the imaging evaluation strictly referred to the international STandards for ReportIng Vascular changes on nEuroimaging (STRIVE) criteria (7). According to the etiological classification of CSVD, all participants were diagnosed with arteriolosclerotic (type 1) CSVD, also defined as age- and vascular risk factor-related CSVD. Patients with other CSVD subtypes, including cerebral amyloid angiopathy, hereditary CSVD, inflammatory vasculopathy, and venous CSVD, were strictly excluded. CSVD diagnosis was comprehensively determined based on typical neuroimaging manifestations, including white matter hyperintensities (WMH), lacunar infarcts, cerebral microbleeds, and enlarged perivascular spaces. All enrolled patients first met the standardized diagnostic criteria for arteriolosclerotic CSVD, and were subsequently divided into WMH-positive and WMH-negative groups according to the presence or absence of WMH. Notably, WMH-negative patients fulfilled the CSVD diagnostic criteria through other typical small-vessel imaging features in the absence of significant WMH lesions.

Among the 382 CSVD patients, there were 184 males (48.2%) and 198 females (51.8%), aged 60–83 years with a mean age of (70.32 ± 5.30) years. Written informed consent was obtained from all patients or their legal representatives prior to study enrollment. This single-center cross-sectional observational study was reported following the STROBE guidelines.

Inclusion criteria: (1) Complete clinical data available; (2) Diagnosis of CSVD consistent with the 2020 Chinese Guidelines for the Diagnosis and Treatment of Cerebral Small-Vessel Disease; (3) Brain magnetic resonance imaging and relevant serological tests completed within 48 h after hospital admission; (4) Patients classified as having the arteriolosclerotic subtype of CSVD; (5) Written informed consent obtained from patients or their family members.

Exclusion criteria: (1) Cardiogenic cerebral infarction caused by rheumatic heart disease, atrial fibrillation, arrhythmia, or other cardiac disorders; (2) Tumor-related stroke, traumatic stroke, or inflammatory stroke; (3) Co-existing cerebrovascular abnormalities including cerebral amyloid angiopathy and Moyamoya disease; (4) Complicated with intracerebral hemorrhage, brain tumor, intracranial infection, demyelinating disease, dementia, or Parkinson’s disease; (5) Presence of severe cardiac, pulmonary, hepatic, or renal diseases, malignancy, severe anemia, or hematological disorders; (6) Use of folic acid, vitamins, or other medications affecting homocysteine metabolism within the previous 3 months; (7) Incomplete clinical data.

### 2.2 Data collection

General information including age, sex, smoking history, drinking history, and medical histories of diabetes mellitus, hypertension, coronary heart disease, and present illness were collected. Auxiliary examinations and laboratory tests were completed within 48 h after admission, including brain MRI + MRA, echocardiography, cervical vascular ultrasound, complete blood count, comprehensive biochemical panel, and other relevant indicators.

### 2.3 Imaging assessment

#### 2.3.1 MRI examination

MRI examinations were performed on a Philips 3.0-T MRI scanner with an 8-channel head phased-array coil. All subjects underwent brain MRI sequences (T1WI, T2WI, T2-FLAIR), DWI, and brain MRA. Scan parameters were as follows: T1WI: TR = 1900 ms, TE = 18 ms; T2WI: TR = 4,000 ms, TE = 110 ms; T2-FLAIR: TI = 2,500 ms, TR = 9,000 ms, TE = 120 ms; DWI: TR = 2,500 ms, TE = 93 ms, slice thickness = 6 mm, slice gap = 1 mm; MRA: TR = 19 ms, TE = 3 ms, slice thickness = 1.6 mm, slice gap = 0.7 mm. All scanning sequences covered the brain from the skull base to the vertex. White-matter lesions were diagnosed based on MRI findings. WMH was defined as hyperintense signals on T2WI and FLAIR, isointense or slightly hypointense signals on T1WI, and without obvious hyperintensity on DWI (DWI was mainly used to exclude patients with acute cerebral infarction). According to the distribution location, WMH was categorized into periventricular white-matter lesions and deep white-matter lesions. The severity of WMH was assessed using the Fazekas scale. ① Periventricular white-matter hyperintensities: 0 = no lesions; 1 = caps or pencil-thin periventricular halo; 2 = smooth periventricular halo; 3 = irregular periventricular hyperintensities extending into the deep white matter. ② Deep white-matter hyperintensities: 0 = no lesions; 1 = punctate foci; 2 = beginning confluence of foci; 3 = large confluent areas. The sum of the two sub-scores yielded the total Fazekas score. Blinded WMH evaluations for brain MRI scans were independently performed by two radiologists with extensive experience in neuro-MRI interpretation. Discrepancies were resolved by consensus between the two radiologists. The weighted Cohen’s kappa coefficient for total Fazekas score was 0.859 (95% CI: 0.792–0.916), demonstrating excellent inter-rater reliability. Subjects were divided into WMH-positive group and WMH-negative group according to the presence or absence of WMH. Based on total Fazekas scores, patients were further stratified by WMH severity: mild WMH group (1–2 points), moderate WMH group (3–4 points), and severe WMH group (5–6 points).

#### 2.3.2 Cervical vascular ultrasonography

Carotid atherosclerotic plaques were detected using a color Doppler ultrasound diagnostic system (Model ARIETTA70, ALOKA, Japan). Patients were placed in the supine position, with the head tilted 45° away from the examiner. Bilateral common carotid arteries, carotid bifurcations, and initial segments of internal and external carotid arteries were scanned sequentially in accordance with the Guidelines for Carotid Ultrasonography. Carotid intima-media thickness (IMT) was defined as the vertical distance from the luminal-intimal interface to the media-adventitia interface. IMT values were documented, and the presence or absence of plaques was identified. Ultrasonographic diagnostic criterion for plaque: IMT ≥ 1.5 mm. The Crouse score was calculated by summing the maximum thickness of each discrete plaque in bilateral carotid arteries.

### 2.4 Blood sample collection and laboratory assays

#### 2.4.1 Measurement of Hcy

Fasting cubital venous blood (5 mL) was collected from participants on the early morning of the second hospital day. Blood was transferred into yellow-top vacuum tubes containing coagulant and separating gel. Samples were allowed to clot fully at room temperature for 30 min, followed by centrifugation at 3000 r/min for 15 min. A 2 mL aliquot of the upper serum was transferred to an EP tube, sequentially coded, and stored frozen at −80 °C until assay. Serum Hcy concentration was measured by professional laboratory technicians of our hospital using a Hitachi 7,600–210 automatic analyzer with the enzymatic cycling assay.

#### 2.4.2 Measurement of SAP and NfL

Fasting cubital venous blood (5 mL) was collected on the early morning of the second hospital day and placed into yellow-top vacuum tubes with coagulant and separating gel. Samples clotted at room temperature for 30–40 min and were centrifuged at 3000 r/min for 15 min at room temperature. A 2 mL aliquot of upper serum was transferred into an EP tube, coded according to study identification numbers, and stored frozen at −80 °C pending analysis. Serum SAP and NfL levels for all participants were quantified by experienced laboratory technicians in our hospital using enzyme-linked immunosorbent assay (ELISA). Commercial ELISA kits were purchased from Jiangsu Baolai Co., Ltd. Assays were performed strictly following the manufacturer’s instructions. The measurable range was 0.2 μg/L – 20 μg/L for SAP and 3 ng/L – 200 ng/L for NfL. Calibration was conducted using kit-supplied calibrators with serial dilution to generate standard curves. Serum samples were diluted 5-fold prior to detection for both biomarkers. All samples were tested in duplicate wells. The intra-assay coefficient of variation (CV) was <8%, and the inter-assay CV was <12%. Laboratory technicians performed assays under blinded conditions with respect to participants’ imaging stratification and clinical baseline data.

#### 2.4.3 Biochemical indicator measurement

Fasting blood glucose, total cholesterol, triglycerides, low-density-lipoprotein cholesterol, high-density-lipoprotein cholesterol, creatinine and other biochemical parameters were determined by professional laboratory technicians using a biochemical analyzer (Beckman Coulter UniCel DxC 800, United States) with matching kits from the Beckman Coulter biochemical analysis system. Fasting blood glucose was measured via the hexokinase method. Low-density-lipoprotein cholesterol and high-density-lipoprotein cholesterol were measured by direct assay. Total cholesterol and creatinine were determined by enzymatic assay. Triglycerides were quantified using the GPO-POD method.

### 2.5 Statistical methods

All study data were entered into Microsoft Excel to establish a database. After strict data verification and cleaning, no missing values existed for all key study variables, and no missing-data handling or imputation was required. Cleaned data were imported into IBM SPSS Statistics for Windows, Version 26.0 (IBM Corporation, Armonk, New York, United States) for statistical analyses. This observational study was reported in accordance with the STROBE (Strengthening the Reporting of Observational Studies in Epidemiology) guidelines.

Normally distributed continuous variables were expressed as mean ± standard deviation ( $\bar{x}$ ± *s*). The independent-samples *t*-test was applied for comparisons of means between two independent groups. Non-normally distributed continuous variables were described as median (interquartile range), and between-group comparisons were performed using the Mann–Whitney *U* test. Categorical variables were presented as number of cases (percentage), and group comparisons were conducted with the Pearson *χ*^2^ test. Fisher’s exact test was adopted when theoretical frequencies failed to meet the requirements for the *χ*^2^ test. Spearman’s rank-order correlation analysis was used for correlation analyses of ordinal categorical variables and non-normally distributed continuous variables. One-way analysis of variance (ANOVA) was used for comparisons among three groups of continuous variables; the Levene’s test was performed to assess homogeneity of variances. Post-hoc pairwise comparisons were conducted using the Tukey HSD test when variances were homogeneous; the Games–Howell test was utilized for heterogeneous variances. Covariate selection for regression models was based on univariate screening; variables with *p* < 0.05 in univariate analyses were entered into binary and ordinal logistic regression models. Sex and serum creatinine were forced into the ordinal logistic regression model as established potential confounders regardless of univariate *p* values. For multivariable analyses, variance inflation factor (VIF) was calculated to assess multicollinearity, and all VIF values were less than 5, indicating no obvious multicollinearity among variables. Binary logistic regression was performed when the dependent variable was dichotomous. For ordinal dependent variables, the proportional-odds assumption was evaluated via the parallel-lines test; the proportional-odds assumption was considered satisfied if the parallel-lines test yielded *p* > 0.05, and ordinal logistic regression was subsequently applied. To control Type I error inflation from multiple statistical tests, false-discovery rate (FDR) correction was applied for binary logistic regression, ordinal logistic regression, and Spearman correlation analyses. Receiver operating characteristic (ROC) curve analysis was regarded as exploratory analysis and interpreted cautiously without additional FDR correction for multiple comparisons, which was used to evaluate the diagnostic performance of serum Hcy, SAP, NfL, and carotid plaque Crouse score for WMH. A two-tailed *p* value < 0.05 was defined as statistically significant.

## 3 Results

### 3.1 Baseline characteristics

Among the 382 patients with CSVD, 232 patients were assigned to the WMH-positive group and 150 to the WMH-negative group. There were no statistically significant differences between the two groups in body mass index (BMI), smoking history, drinking history, history of hypertension, history of diabetes mellitus, history of coronary heart disease, fasting blood glucose, total cholesterol, triglycerides, low-density-lipoprotein cholesterol, high-density-lipoprotein cholesterol, and creatinine (all *p* > 0.05). Statistically significant inter-group differences were observed in male proportion (χ^2^ = 4.620, *p* = 0.036), age (*t* = 8.776, *p <* 0.001), Hcy (*t* = 12.049, *p <* 0.001), SAP (*t* = 15.860, *p <* 0.001), NfL (*t* = 4.224, *p <* 0.001), and carotid plaque Crouse score (*t* = 12.299, *p <* 0.001). All these indicators were higher in the WMH-positive group relative to the WMH-negative group (all *p <* 0.05) (Table 1).

**Table 1:** Comparison of baseline characteristics between the WMH-positive group and the WMH-negative group.

Variable	WMH-positive group (*n* = 232)	WMH-negative group (*n* = 150)	*t/χ^2^*	*p-*value
Male, *n* (%)	122(52.6)	62(41.3)	4.620^a^	0.036
Age (years), $\bar{x}$ ± *s*	72.07 ± 4.83	67.62 ± 4.87	8.776*	<0.001
BMI, $\bar{x}$ ± *s*	26.05 ± 2.07	25.82 ± 2.15	1.061*	0.290
Smoking history, *n* (%)	107(46.1)	66(44.0)	0.165^a^	0.684
Alcohol-drinking history, *n* (%)	95(40.9)	57(38.0)	0.331^a^	0.565
History of hypertension, *n* (%)	134(57.8)	87(58.0)	0.002^a^	0.963
History of diabetes mellitus, *n* (%)	104(44.8)	61(40.7)	0.643^a^	0.423
History of coronary heart disease, *n* (%)	37(15.9)	17(11.3)	1.598^a^	0.206
Fasting blood glucose (mmol/L), $\bar{x}$ ± *s*	8.17 ± 2.97	8.16 ± 3.37	0.030*	0.976
Total cholesterol (mmol/L), $\bar{x}$ ± *s*	4.95 ± 1.60	4.89 ± 1.41	0.342*	0.733
Triglycerides (mmol/L), $\bar{x}$ ± *s*	1.82 ± 1.49	1.78 ± 1.20	0.283*	0.777
Low-density-lipoprotein cholesterol (mmol/L), $\bar{x}$ ± *s*	3.17 ± 1.37	3.00 ± 1.04	1.334*	0.183
High-density-lipoprotein cholesterol (mmol/L), $\bar{x}$ ± *s*	1.28 ± 0.35	1.27 ± 0.34	0.090*	0.928
creatinine (μmol/L), $\bar{x}$ ± *s*	71.31 ± 22.92	67.96 ± 16.48	1.662*	0.097
Hcy (μmol/L), $\bar{x}$ ± *s*	24.28 ± 11.68	13.43 ± 5.79	12.049*	<0.001
SAP (μg/L), $\bar{x}$ ± *s*	8.80 ± 3.23	4.91 ± 1.50	15.860*	<0.001
NfL (ng/L), $\bar{x}$ ± *s*	117.02 ± 35.77	101.02 ± 36.75	4.224*	<0.001
Crouse score, $\bar{x}$ ± *s*	3.06 ± 1.27	1.55 ± 1.11	12.299*	<0.001

BMI, body mass index; Hcy, homocysteine; SAP, serum amyloid P-component; NfL, neurofilament light chain. ^*^t-value from two-sample *t*-test; ^a^*x*^2^-value from chi-square test.

### 3.2 Associations of stratified serum Hcy, SAP, NfL, and carotid plaque Crouse score with WMH

Using the overall mean values of serum Hcy, SAP, NfL, and carotid plaque Crouse score as cutoff points, patients were assigned to the exposed group when each indicator was ≥ its corresponding overall mean value. The *χ*^2^ test was applied to analyze the associations between the above-mentioned indicators and WMH in older adult patients with CSVD. The results demonstrated that serum Hcy, SAP, NfL, and carotid plaque Crouse score were associated with WMH among patients with CSVD (all *p <* 0.05). Benjamini–Hochberg FDR correction was performed for these four tests, and all indicators remained statistically significant after adjustment (FDR-corrected *p* < 0.001). The detection rate of WMH was higher in the high-value groups than in the low-value groups (Table 2). Notably, this mean-based grouping was performed solely for exploratory subgroup comparison. This post-hoc dichotomization is not a validated clinical cutoff, and our primary statistical analyses used biomarker values as continuous variables.

**Table 2:** Association analysis of grouped serum Hcy, SAP, NfL, and carotid plaque Crouse score with WMH in patients with CSVD.

Variable	Level	WMH-positive group (*n* = 232)	WMH-negative group (*n* = 150)	*χ^2^*	*P*-value	*OR*	95%CI
Hcy (μmoL/L)	≥20.02	109	33	24.346	<0.001	3.142	1.975–4.999
	<20.02	123	117				
SAP (μg/L)	≥7.27	124	23	55.905	<0.001	6.340	3.793–10.596
	<7.27	108	127				
NfL (ng/L)	≥110.73	96	35	13.166	<0.001	2.319	1.464–3.673
	<110.73	136	115				
Crouse score (points)	≥2.47	167	19	128.298	<0.001	17.714	10.118–31.012
	<2.47	65	131				

Hcy, homocysteine; SAP, serum amyloid P-component; NfL, neurofilament light chain.

### 3.3 Multivariable binary logistic regression analysis of factors associated with WMH in older adult patients with CSVD

Univariate binary logistic regression was used to screen candidate variables. Variables with *p* < 0.05 in the univariate analysis were entered into the multivariable binary logistic regression model, with the presence of WMH set as the dependent variable (no WMH = 0, WMH present = 1). After adjustment, male sex (*OR* = 1.134, 95% *CI*: 0.543–2.370, *p* = 0.738) and creatinine (*OR* = 0.985, 95% *CI*: 0.967–1.000, *p* = 0.115) were not independently associated with WMH. Age (OR = 1.085, 95% CI: 1.010–1.170, *p* = 0.026), Hcy (OR = 1.219, 95% CI: 1.137–1.310, *p* < 0.001), SAP (OR = 2.496, 95% CI: 1.917–3.250, *p* < 0.001), NfL (OR = 1.015, 95% CI: 1.004–1.030, *p* = 0.010), and Crouse score (OR = 1.819, 95% CI: 1.376–2.400, *p* < 0.001) were independently associated with the presence of WMH in older adult patients with CSVD (Table 3). Benjamini–Hochberg false discovery rate (FDR) correction was applied to the *p*-values derived from this multivariable model, and the above five variables remained statistically significant after FDR correction (*p* < 0.05).

**Table 3:** Univariate and multivariable binary logistic regression analyses of factors associated with the presence of WMH in older adult patients with CSVD.

Variable	Univariable analysis		Multivariable analysis^m^		
	OR (95% CI)	P-value	OR (95% CI)	*P-*value	FDR-corrected *p*-value
Male	1.570 (1.040–2.380)	0.032	1.134 (0.543–2.370)	0.738	0.738
Age	1.200 (1.140–1.260)	<0.001	1.085 (1.010–1.170)	0.026	0.036
BMI	1.054 (0.9561–1.160)	0.289			
Smoking history	1.090 (0.721–1.650)	0.684			
Alcohol-drinking history	1.130 (0.743–1.720)	0.565			
History of hypertension	0.990 (0.653–1.500)	0.963			
History of diabetes mellitus	1.190 (0.782–1.800)	0.423			
History of coronary heart disease	1.480 (0.802–2.750)	0.208			
Fasting blood glucose	1.000 (0.937–1.070)	0.976			
Total cholesterol	1.020 (0.894–1.170)	0.732			
Triglycerides	1.020 (0.879–1.190)	0.776			
Low-density-lipoprotein cholesterol	1.120 (0.946–1.330)	0.185			
High-density-lipoprotein cholesterol	1.030 (0.568–1.860)	0.928			
creatinine	1.014 (1.002–1.030)	0.017	0.985 (0.967–1.000)	0.115	0.134
Hcy	1.193 (1.144–1.245)	<0.001	1.219 (1.137–1.310)	<0.001	<0.001
SAP	2.616 (2.141–3.195)	<0.001	2.496 (1.917–3.250)	<0.001	<0.001
NfL	1.013 (1.007–1.019)	<0.001	1.015 (1.004–1.030)	0.01	0.017
Crouse score	2.721 (2.197–3.370)	<0.001	1.819 (1.376–2.400)	<0.001	<0.001

^m^Variables with *p* < 0.05 in univariate analyses were entered into the multivariable ordinal logistic regression model. Sex and creatinine, as potential confounders, were forced into the model even with *p* > 0.05 in univariate analyses. Benjamini–Hochberg FDR correction was applied to p values of variables in this model. Hcy, homocysteine; SAP, serum amyloid P component; NfL, neurofilament light chain.

### 3.4 Diagnostic performance of each indicator for WMH

Receiver-operating characteristic (ROC) curve analysis was performed to evaluate the diagnostic performance of Hcy, SAP, NfL, carotid plaque Crouse score, and age for WMH lesions in patients with CSVD. All indicators demonstrated diagnostic ability for WMH in patients with CSVD (AUC > 0.500, all *p <* 0.001). SAP yielded the highest diagnostic performance, with an AUC of 0.880, an optimal cutoff value of 6.18 μg/L, a sensitivity of 85.3%, and a specificity of 84.5%. The carotid plaque Crouse score (AUC = 0.822) and serum Hcy (AUC = 0.813) exhibited high specificity yet relatively low sensitivity. Age showed moderate diagnostic performance (AUC = 0.744), whereas serum NfL had the lowest discriminatory capacity (AUC = 0.667). These results suggest that SAP possesses the highest diagnostic value for WMH in patients with CSVD (Table 4 and Figure 1).

**Table 4:** ROC curve analysis of serum Hcy, SAP, NfL, carotid plaque Crouse score and age for diagnosing WMH in patients with CSVD.

Variable	Sensitivity	Specificity	AUC	95%CI	*p-*value
SAP	0.853	0.845	0.88	0.845–0.916	<0.001
NfL	0.841	0.647	0.667	0.609–0.726	<0.001
Hcy	0.453	0.916	0.813	0.77–0.857	<0.001
Crouse score	0.586	0.913	0.822	0.777–0.868	<0.001
Age	0.659	0.793	0.744	0.694–0.795	<0.001

Hcy, homocysteine; SAP, serum amyloid P-component; NfL, neurofilament light chain.

**Figure 1:**
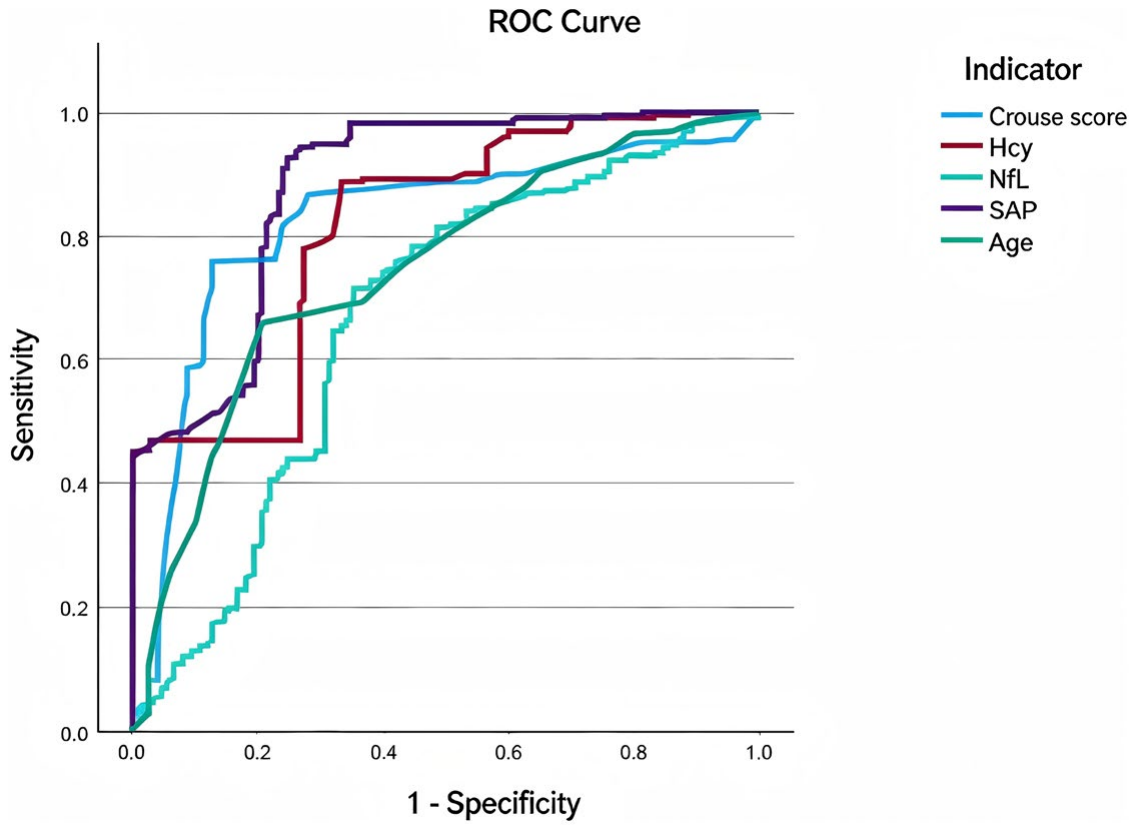
ROC-curve analyses of serum Hcy, SAP, NfL, carotid plaque Crouse score, and age for the diagnosis of WMH.

### 3.5 Comparison of baseline clinical data among CSVD patients with different WMH severity

Among the 232 older adult CSVD patients with WMH, 111 were categorized into the mild WMH group, 71 into the moderate WMH group, and 50 into the severe WMH group. There were no statistically significant differences across the three groups in male proportion, BMI, smoking history, drinking history, history of hypertension, diabetes mellitus, coronary heart disease, fasting blood glucose, total cholesterol, triglycerides, low-density-lipoprotein cholesterol, high-density-lipoprotein cholesterol, and creatinine (all *p* > 0.05). A statistically significant difference in age was detected among the three groups (*F* = 18.964, *p <* 0.001) (Table 5).

**Table 5:** Comparison of baseline clinical data among CSVD patients with different WMH severity.

Variable	Mild WMH group (*n* = 111)	Moderate WMH group (*n* = 71)	Severe WMH group (*n* = 50)	*F/χ* ^2^	*P*-value
Male, *n* (%)	63(56.8)	38(53.5)	21(42.0)	3.047^a^	0.218
Age (years), $\bar{x}$ ± *s*	70.54 ± 4.82	72.24 ± 4.61	75.24 ± 3.41	18.964^b^	< 0.001
BMI, $\bar{x}$ ± *s*	26.15 ± 2.14	26.15 ± 2.06	25.68 ± 1.93	0.999^b^	0.370
Smoking history, *n* (%)	47(42.3)	34(47.9)	26(52.0)	1.422^a^	0.491
Alcohol-drinking history, *n* (%)	45(40.5)	26(36.6)	24(48.0)	1.586^a^	0.452
History of hypertension, *n* (%)	65(58.6)	37(52.1)	32(64.0)	1.755^a^	0.416
History of diabetes mellitus, *n* (%)	48(43.2)	32(45.1)	24(48.0)	0.318^a^	0.853
History of coronary heart disease, *n* (%)	16(14.4)	12(16.9)	9(18.0)	0.400^a^	0.819
Fasting blood glucose (mmol/L), $\bar{x}$ ± *s*	8.40 ± 3.39	7.53 ± 2.37	8.19 ± 2.88	1.854^b^	0.159
Total cholesterol (mmol/L), $\bar{x}$ ± *s*	4.90 ± 1.67	4.51 ± 1.31	5.01 ± 1.46	1.976^b^	0.141
Triglycerides (mmol/L), $\bar{x}$ ± *s*	1.69 ± 1.69	1.88 ± 0.98	1.56 ± 0.97	0.849^b^	0.429
Low-density-lipoprotein cholesterol (mmol/L), $\bar{x}$ ± *s*	3.03 ± 1.21	2.85 ± 0.97	3.07 ± 1.03	0.837^b^	0.434
High-density-lipoprotein cholesterol (mmol/L), $\bar{x}$ ± *s*	1.29 ± 0.32	1.28 ± 0.39	1.25 ± 0.37	0.190^b^	0.827
Serum creatinine (μmol/L), $\bar{x}$ ± *s*	69.76 ± 25.79	75.25 ± 18.42	69.18 ± 21.61	1.528^b^	0.219

BMI, body mass index. ^a^*χ*^2^-value from chi-square test; ^b^*F*-value from one-way analysis of variance.

### 3.6 Comparison of each indicator across different WMH severity groups

One-way analysis of variance (ANOVA) was used to compare serum Hcy, SAP, NfL, and carotid plaque Crouse score among the mild, moderate, and severe WMH groups. Statistically significant overall inter-group differences were observed for all indicators (all *p <* 0.05). Levene’s test for homogeneity of variance indicated that the homogeneity-of-variance assumption was violated for several indicators; therefore, post-hoc pairwise comparisons were conducted using the Games-Howell test. Post-hoc results revealed that levels of all indicators were higher in the moderate and severe WMH groups compared with the mild WMH group, and were higher in the severe WMH group relative to the moderate WMH group; all these inter-group differences reached statistical significance (all *p <* 0.05) (Table 6).

**Table 6:** Comparison of serum Hcy, SAP, NfL levels and carotid plaque Crouse scores among patients with different WMH severity.

Variable	Mild WMH group (*n* = 111)	Moderate WMH group (*n* = 71)	Severe WMH group (*n* = 50)	*F*-value	*P-*value
Hcy (μmol/L)	20.14 ± 8.18	24.20 ± 9.57^c^	33.60 ± 15.31^cd^	18.751	<0.001
SAP (μg/L)	7.72 ± 3.03	8.89 ± 2.30^c^	11.06 ± 3.63^cd^	16.560	<0.001
NfL (ng/L)	104.81 ± 31.15	118.29 ± 30.72^c^	142.31 ± 38.88^cd^	18.575	<0.001
Crouse score (points)	2.62 ± 1.28	3.20 ± 0.75^c^	3.86 ± 1.43^cd^	15.282	<0.001

Results of post-hoc multiple comparisons. ^c^*p* < 0.05 versus the mild WMH group; ^d^*p* < 0.05 versus the moderate WMH group. SAP, serum amyloid P-component; NfL, neurofilament light chain; Hcy, homocysteine.

### 3.7 Correlation between each indicator and WMH severity

Spearman’s rank-order correlation analysis demonstrated that serum Hcy, SAP, NfL, and Crouse score were all significantly positively correlated with WMH severity in patients with CSVD (all *p <* 0.001). After FDR correction for multiple comparisons, all associations remained significant (all FDR-corrected *p* < 0.001). Benjamini–Hochberg FDR correction for multiple comparisons was performed for these four correlation tests; after adjustment, all associations remained statistically significant (all FDR-corrected *p* < 0.001). SAP had the highest correlation coefficient (*r*_s_ = 0.451, 95%CI: 0.339–0.551), followed by Hcy (*r*_s_ = 0.432, 95%CI: 0.318–0.534). NfL (*r*_s_ = 0.389, 95%CI: 0.270–0.496) and Crouse score (*r*_s_ = 0.366, 95%*CI*: 0.246–0.476) showed moderate-strength positive correlations. These findings indicate stable correlations between the four indicators and WMH severity, supporting their potential as biological markers for assessing white-matter lesion severity in older adult patients with CSVD (Table 7).

**Table 7:** Spearman correlation analysis between serum Hcy, SAP, NfL, carotid Crouse score and WMH severity in patients with CSVD.

Variable	*r_s_*	95% confidence interval	*P-*value	FDR-corrected *P*-value
Hcy	0.432	0.318–0.534	<0.001	<0.001
SAP	0.451	0.339–0.551	<0.001	<0.001
NfL	0.389	0.270–0.496	<0.001	<0.001
Crouse score	0.366	0.246–0.476	<0.001	<0.001

Hcy, homocysteine; SAP, serum amyloid P-component; NfL, neurofilament light chain.

### 3.8 Ordinal logistic regression analysis of factors associated with WMH severity in older CSVD patients

Univariate analyses were performed to screen candidate variables. Variables with *p* < 0.05 in univariate analyses were entered into the multivariable ordinal logistic regression model. Sex and creatinine, as recognized potential confounders, were forced into the model despite *p* > 0.05 in univariate testing. WMH severity grading was set as the dependent variable. Multivariable regression showed that sex and creatinine were not independently associated with WMH severity in older adult patients with CSVD. After full adjustment and Benjamini–Hochberg false discovery rate (FDR) correction for multiple testing, age, serum Hcy, SAP, NfL, and carotid Crouse score exhibited an independent positive association with WMH severity (all adjusted *p* < 0.05). The parallel-lines test yielded *p* = 0.56, confirming that the proportional-odds assumption was not violated. The results are shown in Table 8.

**Table 8:** Univariate and multivariable ordinal logistic regression analyses of factors associated with WMH severity in patients with CSVD.

Variable	Univariable analysis		Multivariable analysis^n^		
	OR (95% CI)	*P-*value	OR (95% CI)	*P-*value	FDR-corrected *p*-value
Male	0.676 (0.415–1.10)	0.115	0.685 (0.373–1.250)	0.22	0.2567
Age	1.170 (1.110–1.240)	<0.001	1.163 (1.089–1.250)	<0.001	0.0018
BMI	0.945 (0.835–1.070)	0.366			
Smoking history	1.340 (0.827–2.180)	0.233			
Alcohol-drinking history	1.150 (0.701–1.880)	0.581			
History of hypertension	1.06 (0.651–1.730)	0.817			
History of diabetes mellitus	1.150 (0.704–1.860)	0.584			
History of coronary heart disease	1.230 (0.637–2.360)	0.53			
Fasting blood glucose	0.965 (0.885–1.050)	0.412			
Total cholesterol	0.990 (0.848–1.150)	0.902			
Triglycerides	0.924 (0.774–1.090)	0.359			
Low-density-lipoprotein cholesterol	0.928 (0.775–1.100)	0.402			
High-density-lipoprotein cholesterol	0.829 (0.410–1.630)	0.592			
Serum creatinine	1.000 (0.992–1.010)	0.590	1.001 (0.988–1.010)	0.885	0.885
Hcy	1.090 (1.060–1.120)	<0.001	1.109 (1.076–1.150)	<0.001	0.004
SAP	1.310 (1.200–1.430)	<0.001	1.133 (1.021–1.260)	0.019	0.027
NfL	1.020 (1.020–1.030)	<0.001	1.024 (1.014–1.030)	<0.001	0.007
Crouse score	2.070 (1.630–2.670)	<0.001	2.157 (1.602–2.980)	<0.001	0.002

^n^Variables with *p* < 0.05 in univariate analyses were included in the multivariable ordinal logistic regression model. Sex and creatinine were forced into the model as potential confounders, even though they had *p* > 0.05 in univariate analyses. The Benjamini–Hochberg method was applied for false discovery rate (FDR) correction of the *p*-values of variables within this multivariable model. BMI, body mass index; Hcy, homocysteine; SAP, serum amyloid P component; NfL, neurofilament light chain.

## 4 Discussion

This study enrolled older adult patients with CSVD to analyze the relationships of serum Hcy, SAP, NfL, and carotid plaque Crouse score with the occurrence and severity of WMH. Univariate analyses revealed that all observed indicators were significantly higher in the WMH-positive group than in the WMH-negative group. Binary logistic regression was performed with the presence versus absence of WMH as the dichotomous outcome. After mutual adjustment for confounding factors among variables within the regression model, all the above-mentioned indicators were independently correlated with WMH occurrence; elevated indicator levels were associated with higher odds of prevalent WMH, with SAP exhibiting the most prominent association effect. Spearman correlation analysis demonstrated that these indicators were significantly positively correlated with WMH severity. Ordinal logistic regression, using graded WMH severity as the ordinal outcome, further showed independent associations between each indicator and WMH lesion severity. The proportional-odds assumption was validated by the parallel-lines test, supporting the reliability of this ordinal model; higher biomarker levels were associated with greater odds of more severe WMH, and the ORs reflected the magnitude of these cross-sectional associations. Indicator levels increased as WMH severity worsened and were positively correlated with white-matter lesion burden. ROC analyses indicated that age, serum Hcy, SAP, NfL, and carotid plaque Crouse score all possessed certain clinical diagnostic performance for WMH. Serum SAP yielded the optimal diagnostic value and may serve as a reliable auxiliary indicator for evaluating WMH burden and lesion severity in older adult patients with CSVD.

Both univariate and multivariable binary and ordinal regression analyses in the present study showed that age was independently associated with WMH presence and severity; older patients presented with more severe WMH lesions, suggesting that age exhibits an independent association with WMH occurrence and high WMH burden among older adult CSVD patients. This finding is consistent with previous studies. An Asian-population study (8) reported that WMH progresses with advancing age. Several longitudinal studies have also confirmed that age can predict WMH progression, and increased age is closely linked to higher risk of incident WMH, faster progression, and greater magnitude of lesion volume change (9). Existing evidence indicates that age is related to white-matter injury mainly through two core pathways: myelin degenerative changes and deterioration of cerebral small-vessel function. On one hand, cerebral white matter is predominantly composed of myelinated nerve fibers. With aging, the contents of key structural components such as myelin basic protein decrease, resulting in loosening and loss of myelin micro-architecture, disruption of nerve-fiber structural integrity, and ultimately abnormal WMH signals on neuroimaging (10, 11). Aging-related myelin degeneration acts independent of conventional vascular risk factors and represents an important intrinsic mechanism for age-related white-matter damage. On the other hand, aging induces cerebral small-artery atherosclerosis, leading to increased vascular wall stiffness and reduced vascular compliance, which markedly impair cerebral autoregulation and hemodynamic compensatory capacity (12). Under hemodynamic disturbances such as blood-pressure fluctuations, deep cerebral white-matter regions are susceptible to insidious, recurrent chronic hypoperfusion. Sustained ischemic–hypoxic injury disrupts white-matter fiber architecture and further correlates with WMH genesis and aggravation (8, 13). Taken together, advancing age shows a robust independent association with WMH occurrence and increased severity. Nevertheless, age is irreversible and cannot be modified by clinical interventions. Therefore, clinical prevention of white-matter lesions cannot rely on age-targeted strategies. Instead, attention should be directed toward other modifiable risk factors and underlying mechanisms to explore effective interventions for delaying the onset and progression of white-matter injury in older adults.

At present, conclusions regarding the association between sex and WMH remain inconsistent (14–16). Existing studies show substantial heterogeneity: several investigations have reported significantly higher WMH burden in female subjects than in male subjects (14, 17–19), whereas multiple other studies have identified no definite correlation between sex and WMH onset (15, 20). Such discrepancies in research findings are mainly modulated by multiple factors including study-cohort sources, racial-ethnic differences among populations, and the completeness of confounding-factor adjustment (21–23). The present study enrolled hospitalized older adult patients. Univariate analysis demonstrated a higher proportion of male patients in the WMH-positive group relative to the WMH-negative group. Nevertheless, after adjusting for relevant confounders, binary and ordinal logistic regression analyses indicated that sex was not independently associated with either WMH occurrence or WMH severity among hospitalized older adults. This finding is consistent with results from several previous cohort studies (15, 23). Traditional mechanistic viewpoints postulate that the sharp decline in estrogen levels after female menopause diminishes the protective effects on cerebral vascular endothelium and cerebral myelin, which may elevate the risk of WMH. However, multiple well-matched large-sample studies in recent years have challenged this notion, confirming that menopause is not an independent driving factor for increased WMH burden and that confounding effects attributed to age constitute the core cause of spurious associations between sex and WMH (21). Cumulative evidence has demonstrated prominent sex-specific effects of vascular risk factors on white-matter injury. Under comparable conditions of abnormal blood pressure and diabetes exposure, cerebral small vessels in females are more vulnerable to damage, thereby triggering WMH lesions (18, 20). Meanwhile, obvious sex-related differences exist in the pathological features of WMH across distinct brain regions as well as in pathways whereby obesity mediates WMH development (19, 23). These sex-specific pathological effects are readily masked by confounders such as age and underlying comorbidities, which reasonably explains why no independent association between sex and WMH was observed after adjustment for confounding variables in the present study. Furthermore, notable discrepancies exist between findings derived from hospitalized-patient cohorts and those from community-based general populations. This further suggests that future CSVD-related studies should expand population coverage to include community-dwelling participants. Meanwhile, sex should be treated as an effect modifier for stratified analyses to minimize result bias induced by confounding factors (22). The absence of independent associations between sex and either WMH occurrence or severity in our study may be attributable to several limitations. Based on a single hospitalized-patient cohort, this work carries certain selection bias. Moreover, given our limited sample size and the fact that all participants were of East-Asian ancestry, the statistical power was insufficient to detect weak sex-related association effects.

Hypertension, diabetes mellitus, and dyslipidemia are classic risk factors for CSVD; they can accelerate intracerebral small-artery hyalinosis and promote white-matter injury (22, 23). Nevertheless, no inter-group differences in these traditional metabolic risk factors were observed in the present study. This indicates that marked white-matter damage can still develop in some older adult CSVD patients even without typical metabolic disturbances. It suggests that vascular inflammation, axonal injury, and extracranial atherosclerotic burden represent important pathogenic pathways independent of conventional risk factors (24–26). Hcy is a sulfur-containing intermediate metabolite derived from methionine metabolism and is regarded as an inflammatory biomarker associated with endothelial injury. Insufficient intake or impaired metabolism of folate and vitamin B12 blocks the remethylation pathway of Hcy, leading to its accumulation and elevation in peripheral blood, which participates in diverse pathological processes of systemic and cerebral microvascular endothelial injury (27, 28). A community-based cross-sectional study conducted by Zhang et al. (29) demonstrated that serum Hcy was significantly associated with cerebral small-vessel injury after adjustment for confounders including age, blood pressure, blood glucose, and blood lipids. In the present study, univariate analysis, multivariable binary and ordinal logistic regression, and Spearman correlation analysis consistently demonstrated that serum Hcy levels were independently correlated with both WMH occurrence and severity; serum Hcy increased as WMH severity worsened, indicating that elevated Hcy is independently correlated with aggravated WMH burden in older adult patients with CSVD. These findings are consistent with multiple previous reports (3, 29–31). Hcy mediates white-matter lesions mainly through dual pathways of endothelial injury and chronic inflammation. High Hcy concentrations activate oxidative-stress pathways, directly damaging cerebral microvascular endothelium, inhibiting nitric-oxide production, impairing vasodilatory function, and inducing persistent cerebral white-matter hypoperfusion (32). Meanwhile, Hcy up-regulates vascular cell adhesion molecule-1 (VCAM-1) and other vascular adhesion molecules, promoting leukocyte adhesion to vascular walls and massive release of pro-inflammatory mediators, which triggers sustained perivascular inflammation. Such inflammatory injury impairs oligodendrocyte function, inhibits myelin synthesis, induces demyelination and disorganized axonal arrangement, and ultimately results in structural damage to cerebral white matter (29). In addition, abnormally elevated Hcy accelerates lipohyalinosis of cerebral small arteries, giving rise to luminal stenosis and further reduction in cerebral perfusion, which progressively aggravates ischemic white-matter injury and finally manifests as white-matter hyperintensities on FLAIR MRI sequences. Notably, these pathological mechanisms are derived from preclinical evidence and previous translational studies; the present cross-sectional data cannot confirm causal or modifying effects of Hcy on WMH progression. In combination with the above pathological mechanisms and our study findings, serum Hcy levels are statistically associated with cerebral small-vessel injury as well as the incidence and severity of WMH among CSVD patients, supporting its potential biomarker value in reflecting WMH lesion status. ROC curve analysis in this study yielded an AUC of 0.813 for Hcy in diagnosing WMH, with acceptable specificity yet relatively low sensitivity. This indicates that Hcy can serve as an auxiliary diagnostic marker for WMH. Nevertheless, owing to its insufficient sensitivity, standalone Hcy testing for early WMH screening carries a risk of missed diagnosis. Clinically, combined assessment with other indicators is required to improve screening and diagnostic performance. Notably, a recent study by Liu et al. (2) described heterogeneous effects of Hcy and Crouse score on periventricular versus deep subcortical WMH stratified by anatomical location. Unlike their location-specific analysis, our work focused on global WMH occurrence and severity and further incorporated SAP and NfL, providing additional evidence from multiple circulating biomarkers beyond Hcy and carotid plaque burden.

The SAP is synthesized by the liver. Under physiological conditions, the blood–brain barrier blocks peripheral SAP from entering the central nervous system, and the cerebrospinal fluid SAP concentration is only one-thousandth of that in plasma (33). Pathological conditions such as aging, traumatic brain injury, intracerebral hemorrhage, and vascular damage disrupt blood–brain-barrier integrity, allowing massive leakage of peripheral plasma SAP into the brain parenchyma and increasing SAP exposure within brain tissue. Existing in-vitro cellular and animal experiments (34–37) have demonstrated that SAP exerts direct neurotoxicity, which can damage neurons and induce neuronal apoptosis, and this toxic effect can act independently of β-amyloid protein (Aβ) (38). There is abundant research concerning SAP in Alzheimer’s disease; nevertheless, clinical studies exploring the association between SAP and white-matter hyperintensities among CSVD populations remain scarce (39). The present study, based on univariate, multivariable binary and ordinal logistic regression analyses, revealed that SAP was independently associated with the occurrence and severity of WMH, and SAP levels increased as WMH lesions deteriorated. These findings indicate that SAP shows a strong independent correlation with WMH onset and severe lesions in older adult patients with CSVD. Among all indicators included in this study, SAP exhibited the most prominent association effect, which was independent of conventional vascular risk factors such as hypertension and diabetes mellitus. This suggests that the amyloid-protein pathway may be closely involved in substantial white-matter injury occurring in older adults even in the absence of typical metabolic disturbances, which is consistent with conclusions from a two-sample Mendelian randomization study reported by Wang et al. (40). The potential pathological mechanisms whereby SAP participates in white-matter injury can be summarized as follows: cerebral small-vessel endothelial damage abnormally elevates blood–brain-barrier permeability, and large amounts of peripheral SAP infiltrate the perivascular tissues of cerebral white matter (41). On the one hand, SAP can bind to and stabilize Aβ amyloid fibrils, inhibiting Aβ degradation and clearance and aggravating cerebral amyloid deposition (38, 42). On the other hand, SAP triggers chronic perivascular inflammatory responses, activates microglia, and induces robust release of pro-inflammatory cytokines. This process damages oligodendrocytes, causes demyelination, axonal fragmentation, and disorganization of white-matter fiber tracts, ultimately producing WMH lesions on MRI (39, 40). Using human post-mortem traumatic-brain-injury specimens, Yip et al. (41) further confirmed that disruption of blood–brain-barrier integrity enables peripheral SAP to specifically invade injured neuronal axons and accumulate persistently. Based on preclinical evidence, SAP is proposed to mediate white-matter degenerative lesions through dual pathways: disruption of the neurofilament cytoskeleton and promotion of Aβ amyloid deposition. ROC-curve analyses in the current study demonstrated that SAP yielded the best diagnostic performance for WMH (AUC = 0.880; optimal cutoff value, 6.18 μg/L; sensitivity, 85.3%; specificity, 84.5%). This finding suggests that routine clinical monitoring of serum SAP levels can effectively assist early identification of WMH lesions and evaluation of lesion severity, providing important evidence for early intervention in older adult patients with CSVD.

NfL is a core protein of the axonal cytoskeleton responsible for maintaining axonal structural stability and regulating axonal diameter, and it is exclusively localized within neuronal axons (43–45). It is barely released into the extracellular space when axons remain intact (46–48). Once axonal injury, degeneration, or necrosis occurs and cell-membrane integrity is disrupted, large quantities of intracellular NfL are released into the brain interstitium, diffuse into cerebrospinal fluid, and cross the blood–brain barrier to enter peripheral blood (44–46, 49). Therefore, serum NfL concentration may reflect the overall burden of brain axonal injury, but it lacks disease specificity. Accordingly, serum NfL concentration can directly reflect the total burden of axonal injury within the brain. A study by Chong et al. (43) confirmed that circulating NfL is a specific serum biomarker for neuronal axonal injury and can reflect the severity of axonal injury; diverse pathological insults to the central nervous system can trigger its elevation. Neurological disorders characterized by neuronal-axonal destruction, including Alzheimer’s disease, multiple sclerosis, traumatic brain injury, and frontotemporal dementia, all lead to increased serum NfL levels (46). Notably, serum NfL only quantifies the severity of axonal damage and cannot discriminate whether the injury originates from cerebral small-vessel disease or neurodegenerative disease; etiological diagnosis must rely on complementary MRI findings. CSVD affects intracerebral small arteries and capillaries. Via multiple mechanisms including chronic cerebral ischemia, microbleeds, and persistent perivascular inflammation, CSVD induces sustained axonal damage and subsequent substantial NfL release (50, 51). A multi-ethnic older-adult cohort study by Lockhart et al. (52) demonstrated that plasma NfL was independently positively correlated with WMH burden and white-matter free-water content after adjustment for age-related, metabolic, and renal confounders, supporting plasma NfL as a reliable peripheral biomarker for axonal injury in CSVD. A longitudinal cohort study performed by Qu et al. (44) drew similar conclusions: among cognitively unimpaired older adults, baseline plasma NfL was significantly positively correlated with overall CSVD burden, white-matter hyperintensities, and lacunar infarcts, and may reflect static lesion severity of CSVD Longitudinal follow-up further revealed that the rate of plasma-NfL elevation independently predicted long-term progression risk of global CSVD burden and white-matter lesions. These previous findings indicate that NfL serves as a biomarker of axonal injury rather than a pathogenic mediator of white-matter lesions. In the present study, univariate analysis, multivariable binary and ordinal logistic regression analyses consistently demonstrated that serum NfL was independently associated with both WMH occurrence and severity; higher NfL levels corresponded to more severe white-matter damage in older adult CSVD patients. These results are consistent with multiple prior investigations (47–52). Egle et al. (53) put forward supplementary viewpoints regarding the clinical utility of NfL: although baseline NfL can predict long-term WMH progression in CSVD patients, 3-year follow-up demonstrated minimal dynamic fluctuations of NfL over the disease course. This suggests that NfL is not suitable as a surrogate endpoint for clinical drug trials, and changes in NfL alone cannot be used to evaluate therapeutic responses to pharmacological interventions. ROC-curve analysis in our study showed modest discriminatory ability of serum NfL for predicting WMH in older adult CSVD patients (AUC = 0.667). While this performance is insufficient for standalone clinical diagnosis, it still demonstrates a meaningful association between serum NfL and WMH among hospitalized older patients with CSVD. The suboptimal diagnostic performance may be partly attributable to extracranial sources of NfL, heterogeneous transport across the blood–brain barrier, and the single-center cross-sectional design of the present study. Its clinical performance requires further validation by large-sample, multicenter prospective cohort studies.

Carotid atherosclerosis constitutes an important associated marker for CSVD. It mediates white-matter injury and triggers WMH through dual mechanisms of chronic cerebral hypoperfusion and microembolism. Cerebral white-matter regions are mainly supplied by end-arteries with poor collateral-circulation compensation. Carotid atherosclerotic plaques can produce sustained insufficiency of intracranial blood perfusion. Long-term chronic hypoperfusion damages the endothelium of cerebral microvessels and disrupts blood–brain-barrier integrity, thereby inducing demyelinating injury (54). Meanwhile, microemboli shed from unstable carotid plaques can occlude terminal microvessels within the cerebral white matter. Repeated microinfarcts, together with microvascular injury and blood–brain-barrier disruption mediated by inflammatory factors, jointly induce myelin and axonal damage and eventually lead to intracranial WMH lesions (6, 55, 56). The carotid plaque Crouse score is a reliable metric for quantifying the overall burden of systemic carotid atherosclerosis; higher scores indicate more severe carotid atherosclerotic disease and elevated likelihood of vascular injury. Early previous studies (57) proposed that white-matter lesions originated predominantly from intrinsic cerebral small-vessel pathology, and extracranial carotid atherosclerosis was not regarded as a major driver of CSVD. Nevertheless, accumulating recent evidence suggests that extracranial large-vessel disease serves as an important associated marker for CSVD. A large-sample cross-sectional study by Zhang et al. (58) among asymptomatic Asian participants demonstrated that the carotid Crouse score was independently associated with WMH incidence after adjustment for confounders including age, blood pressure, and metabolic parameters. A dose-dependent relationship existed, whereby higher carotid atherosclerotic burden corresponded to greater likelihood of white-matter injury. A secondary analysis of a community-based cohort with more than 20-year follow-up (59) further validated that baseline carotid stiffness could predict long-term WMH burden. Reduced peripheral large-artery elasticity mediates cerebral small-vessel injury and participates in the pathogenesis of WMH. In the present study, univariate analysis, multivariable binary and ordinal logistic regression analyses, and Spearman correlation analysis consistently demonstrated that the carotid Crouse score was independently associated with both WMH occurrence and severity; higher Crouse scores were accompanied by more severe WMH lesions in older adult CSVD patients. These findings indicate that high carotid atherosclerotic burden is an independent correlate for severe WMH in older adult CSVD patients, consistent with multiple earlier reports (60–62). A retrospective cohort study by Liu et al. (2) also confirmed that, after multivariable adjustment, the carotid Crouse score represented a shared independent correlate for both periventricular and deep-cortical WMH in CSVD patients. Collectively, these studies verify that the carotid plaque Crouse score is a valuable indicator for predicting the occurrence, severity, and anatomical distribution of WMH in CSVD patients. Early clinical interventions targeting anti-atherosclerosis and plaque stabilization may help mitigate white-matter injury burden, though such protective effects cannot be confirmed by our cross-sectional data. Furthermore, measurement of the carotid Crouse score features non-invasiveness, convenience, and favorable reproducibility, making it a promising practical imaging marker for rapid clinical evaluation and risk prediction of white-matter injury. ROC-curve analyses in our study revealed that the carotid Crouse score exhibited high specificity yet relatively low sensitivity for predicting WMH among CSVD patients. This observation can be explained by several factors. First, WMH pathogenesis is multifactorial. Multiple pathways including intrinsic cerebral small-vessel disease, metabolic disturbances, and amyloid-protein-mediated injury can independently trigger white-matter damage without relying on carotid atherosclerosis, which reduces the screening sensitivity of the Crouse score. Second, high carotid-plaque burden is frequently accompanied by long-standing systemic vascular damage, conferring a high probability of concurrent white-matter injury and few clinical false-positive cases, hence the favorable specificity. Third, the Crouse score quantifies only plaque thickness and overall plaque burden; it cannot reflect plaque stability or the risk of microembolus shedding. Such limited evaluation dimensions further constrain its screening sensitivity.

### 4.1 Strengths and limitations

This study simultaneously enrolled serum Hcy, SAP, NfL, and carotid plaque Crouse score to systematically investigate their correlations and independent effects on WMH lesions in older-adult patients with CSVD from multiple dimensions including metabolism, inflammation, axonal injury, and large-vessel disease. The clinical diagnostic performance of these indicators for WMH was also analyzed. Our results demonstrated that all the above-mentioned indicators possessed diagnostic performance for WMH in patients with CSVD, with SAP showing the highest diagnostic value. Clinical studies exploring the association between SAP and WMH remain limited, and the findings of the present study may provide clinical evidence for this research field.

This work has several limitations. First, all participants were hospitalized older-adult patients, leading to selection bias; therefore, the generalizability of our conclusions to community-dwelling older adults is restricted. Second, only peripheral serum biomarkers were measured without cerebrospinal-fluid (CSF) samples. CSF NfL better reflects central axonal injury, and biomarker transport across the blood–brain barrier is variable. Since SAP is mainly hepatically synthesized, serum levels may not accurately represent cerebral neuroinflammation. Thus, interpretation of serum NfL and SAP results requires caution, as we cannot directly capture local brain pathological changes. Third, this is a single-center cross-sectional study. All analyses only identified statistical associations between variables, and definitive causal relationships or temporal sequences cannot be established. No directional or causal conclusion should be inferred from the present findings. Residual confounding may still exist and could affect our observed associations.

## 5 Conclusion

In this single-center cross-sectional study, serum Hcy, SAP, NfL, and carotid plaque Crouse score were independently correlated with the occurrence and severity of WMH in older-adult patients with CSVD, with SAP exhibiting the most prominent association effect. The levels of these biomarkers increased with worsening WMH severity and were positively correlated with lesion burden. They may serve as auxiliary clinical assessment indicators for identifying populations with a higher likelihood of WMH and evaluating lesion severity. No causal inference or recommendations for therapeutic intervention can be drawn from these cross-sectional data.

## Data Availability

The raw data supporting the conclusions of this article will be made available by the authors, without undue reservation.
